# Deep Learning for Computer Vision: A Brief Review

**DOI:** 10.1155/2018/7068349

**Published:** 2018-02-01

**Authors:** Athanasios Voulodimos, Nikolaos Doulamis, Anastasios Doulamis, Eftychios Protopapadakis

**Affiliations:** ^1^Department of Informatics, Technological Educational Institute of Athens, 12210 Athens, Greece; ^2^National Technical University of Athens, 15780 Athens, Greece

## Abstract

Over the last years deep learning methods have been shown to outperform previous state-of-the-art machine learning techniques in several fields, with computer vision being one of the most prominent cases. This review paper provides a brief overview of some of the most significant deep learning schemes used in computer vision problems, that is, Convolutional Neural Networks, Deep Boltzmann Machines and Deep Belief Networks, and Stacked Denoising Autoencoders. A brief account of their history, structure, advantages, and limitations is given, followed by a description of their applications in various computer vision tasks, such as object detection, face recognition, action and activity recognition, and human pose estimation. Finally, a brief overview is given of future directions in designing deep learning schemes for computer vision problems and the challenges involved therein.

## 1. Introduction

Deep learning allows computational models of multiple processing layers to learn and represent data with multiple levels of abstraction mimicking how the brain perceives and understands multimodal information, thus implicitly capturing intricate structures of large‐scale data. Deep learning is a rich family of methods, encompassing neural networks, hierarchical probabilistic models, and a variety of unsupervised and supervised feature learning algorithms. The recent surge of interest in deep learning methods is due to the fact that they have been shown to outperform previous state-of-the-art techniques in several tasks, as well as the abundance of complex data from different sources (e.g., visual, audio, medical, social, and sensor).

The ambition to create a system that simulates the human brain fueled the initial development of neural networks. In 1943, McCulloch and Pitts [1] tried to understand how the brain could produce highly complex patterns by using interconnected basic cells, called neurons. The McCulloch and Pitts model of a neuron, called a MCP model, has made an important contribution to the development of artificial neural networks. A series of major contributions in the field is presented in Table 1, including LeNet [2] and Long Short-Term Memory [3], leading up to today's “era of deep learning.” One of the most substantial breakthroughs in deep learning came in 2006, when Hinton et al. [4] introduced the Deep Belief Network, with multiple layers of Restricted Boltzmann Machines, greedily training one layer at a time in an unsupervised way. Guiding the training of intermediate levels of representation using unsupervised learning, performed locally at each level, was the main principle behind a series of developments that brought about the last decade's surge in deep architectures and deep learning algorithms.

Among the most prominent factors that contributed to the huge boost of deep learning are the appearance of large, high-quality, publicly available labelled datasets, along with the empowerment of parallel GPU computing, which enabled the transition from CPU-based to GPU-based training thus allowing for significant acceleration in deep models' training. Additional factors may have played a lesser role as well, such as the alleviation of the vanishing gradient problem owing to the disengagement from saturating activation functions (such as hyperbolic tangent and the logistic function), the proposal of new regularization techniques (e.g., dropout, batch normalization, and data augmentation), and the appearance of powerful frameworks like TensorFlow [5], theano [6], and mxnet [7], which allow for faster prototyping.

Deep learning has fueled great strides in a variety of computer vision problems, such as object detection (e.g., [8, 9]), motion tracking (e.g., [10, 11]), action recognition (e.g., [12, 13]), human pose estimation (e.g., [14, 15]), and semantic segmentation (e.g., [16, 17]). In this overview, we will concisely review the main developments in deep learning architectures and algorithms for computer vision applications. In this context, we will focus on three of the most important types of deep learning models with respect to their applicability in visual understanding, that is, Convolutional Neural Networks (CNNs), the “Boltzmann family” including Deep Belief Networks (DBNs) and Deep Boltzmann Machines (DBMs) and Stacked (Denoising) Autoencoders. Needless to say, the current coverage is by no means exhaustive; for example, Long Short-Term Memory (LSTM), in the category of Recurrent Neural Networks, although of great significance as a deep learning scheme, is not presented in this review, since it is predominantly applied in problems such as language modeling, text classification, handwriting recognition, machine translation, speech/music recognition, and less so in computer vision problems. The overview is intended to be useful to computer vision and multimedia analysis researchers, as well as to general machine learning researchers, who are interested in the state of the art in deep learning for computer vision tasks, such as object detection and recognition, face recognition, action/activity recognition, and human pose estimation.

The remainder of this paper is organized as follows. In Section 2, the three aforementioned groups of deep learning model are reviewed: Convolutional Neural Networks, Deep Belief Networks and Deep Boltzmann Machines, and Stacked Autoencoders. The basic architectures, training processes, recent developments, advantages, and limitations of each group are presented. In Section 3, we describe the contribution of deep learning algorithms to key computer vision tasks, such as object detection and recognition, face recognition, action/activity recognition, and human pose estimation; we also provide a list of important datasets and resources for benchmarking and validation of deep learning algorithms. Finally, Section 4 concludes the paper with a summary of findings.

## 2. Deep Learning Methods and Developments

### 2.1. Convolutional Neural Networks

Convolutional Neural Networks (CNNs) were inspired by the visual system's structure, and in particular by the models of it proposed in [18]. The first computational models based on these local connectivities between neurons and on hierarchically organized transformations of the image are found in Neocognitron [19], which describes that when neurons with the same parameters are applied on patches of the previous layer at different locations, a form of translational invariance is acquired. Yann LeCun and his collaborators later designed Convolutional Neural Networks employing the error gradient and attaining very good results in a variety of pattern recognition tasks [20–22].

**Figure 1:**
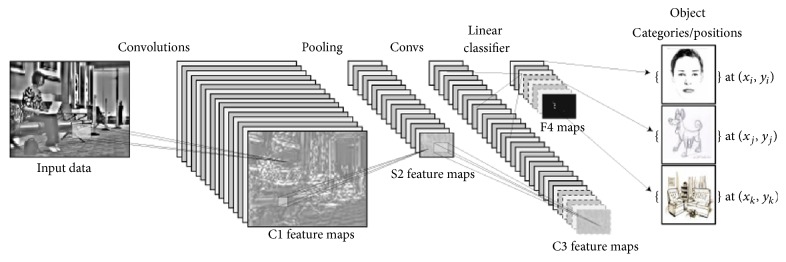
Example architecture of a CNN for a computer vision task (object detection).

A CNN comprises three main types of neural layers, namely, (i) convolutional layers, (ii) pooling layers, and (iii) fully connected layers. Each type of layer plays a different role.  Figure 1 shows a CNN architecture for an object detection in image task. Every layer of a CNN transforms the input volume to an output volume of neuron activation, eventually leading to the final fully connected layers, resulting in a mapping of the input data to a 1D feature vector. CNNs have been extremely successful in computer vision applications, such as face recognition, object detection, powering vision in robotics, and self-driving cars.

*(i) Convolutional Layers.* In the convolutional layers, a CNN utilizes various kernels to convolve the whole image as well as the intermediate feature maps, generating various feature maps. Because of the advantages of the convolution operation, several works (e.g., [23, 24]) have proposed it as a substitute for fully connected layers with a view to attaining faster learning times.

*(ii) Pooling Layers.* Pooling layers are in charge of reducing the spatial dimensions (width × height) of the input volume for the next convolutional layer. The pooling layer does not affect the depth dimension of the volume. The operation performed by this layer is also called subsampling or downsampling, as the reduction of size leads to a simultaneous loss of information. However, such a loss is beneficial for the network because the decrease in size leads to less computational overhead for the upcoming layers of the network, and also it works against overfitting. Average pooling and max pooling are the most commonly used strategies. In [25] a detailed theoretical analysis of max pooling and average pooling performances is given, whereas in [26] it was shown that max pooling can lead to faster convergence, select superior invariant features, and improve generalization. Also there are a number of other variations of the pooling layer in the literature, each inspired by different motivations and serving distinct needs, for example, stochastic pooling [27], spatial pyramid pooling [28, 29], and def-pooling [30].

*(iii) Fully Connected Layers.* Following several convolutional and pooling layers, the high-level reasoning in the neural network is performed via fully connected layers. Neurons in a fully connected layer have full connections to all activation in the previous layer, as their name implies. Their activation can hence be computed with a matrix multiplication followed by a bias offset. Fully connected layers eventually convert the 2D feature maps into a 1D feature vector. The derived vector either could be fed forward into a certain number of categories for classification [31] or could be considered as a feature vector for further processing [32].

The architecture of CNNs employs three concrete ideas: (a) local receptive fields, (b) tied weights, and (c) spatial subsampling. Based on local receptive field, each unit in a convolutional layer receives inputs from a set of neighboring units belonging to the previous layer. This way neurons are capable of extracting elementary visual features such as edges or corners. These features are then combined by the subsequent convolutional layers in order to detect higher order features. Furthermore, the idea that elementary feature detectors, which are useful on a part of an image, are likely to be useful across the entire image is implemented by the concept of tied weights. The concept of tied weights constraints a set of units to have identical weights. Concretely, the units of a convolutional layer are organized in planes. All units of a plane share the same set of weights. Thus, each plane is responsible for constructing a specific feature. The outputs of planes are called feature maps. Each convolutional layer consists of several planes, so that multiple feature maps can be constructed at each location.

During the construction of a feature map, the entire image is scanned by a unit whose states are stored at corresponding locations in the feature map. This construction is equivalent to a convolution operation, followed by an additive bias term and sigmoid function:

(1) $$\begin{array}{c} y^{\left(d\right)}=\sigma \left(\;Wy^{\left(d-1\right)}+b\;\right), \end{array}$$

where *d* stands for the depth of the convolutional layer, **W** is the weight matrix, and **b** is the bias term. For fully connected neural networks, the weight matrix is full, that is, connects every input to every unit with different weights. For CNNs, the weight matrix **W** is very sparse due to the concept of tied weights. Thus, **W** has the form of

(2) $$\begin{array}{c} \left[\begin{array}{cccc} w & 0 & \cdots & 0\\ 0 & w & \cdots & 0\\ \vdots & \cdots & \ddots & \vdots \\ 0 & \cdots & 0 & w \end{array}\right], \end{array}$$

where **w** are matrices having the same dimensions with the units' receptive fields. Employing a sparse weight matrix reduces the number of network's tunable parameters and thus increases its generalization ability. Multiplying **W** with layer inputs is like convolving the input with **w**, which can be seen as a trainable filter. If the input to *d* − 1 convolutional layer is of dimension *N* × *N* and the receptive field of units at a specific plane of convolutional layer *d* is of dimension *m* × *m*, then the constructed feature map will be a matrix of dimensions (*N* − *m* + 1)×(*N* − *m* + 1). Specifically, the element of feature map at (*i*, *j*) location will be

(3) $$\begin{array}{c} y_{ij}^{\left(d\right)}=\sigma \left(\;x_{ij}^{\left(d\right)}+b\;\right) \end{array}$$

with

(4) $$\begin{array}{c} x_{ij}^{\left(d\right)}=\overset{m-1}{\underset{\alpha =0}{\sum }}\;\overset{m-1}{\underset{b=0}{\sum }}w_{\alpha b}y_{\left(i+\alpha \right)\left(j+b\right)}^{\left(d-1\right)}, \end{array}$$

where the bias term *b* is scalar. Using (4) and (3) sequentially for all (*i*, *j*) positions of input, the feature map for the corresponding plane is constructed.

One of the difficulties that may arise with training of CNNs has to do with the large number of parameters that have to be learned, which may lead to the problem of overfitting. To this end, techniques such as stochastic pooling, dropout, and data augmentation have been proposed. Furthermore, CNNs are often subjected to pretraining, that is, to a process that initializes the network with pretrained parameters instead of randomly set ones. Pretraining can accelerate the learning process and also enhance the generalization capability of the network.

Overall, CNNs were shown to significantly outperform traditional machine learning approaches in a wide range of computer vision and pattern recognition tasks [33], examples of which will be presented in Section 3. Their exceptional performance combined with the relative easiness in training are the main reasons that explain the great surge in their popularity over the last few years.

### 2.2. Deep Belief Networks and Deep Boltzmann Machines

**Figure 2:**
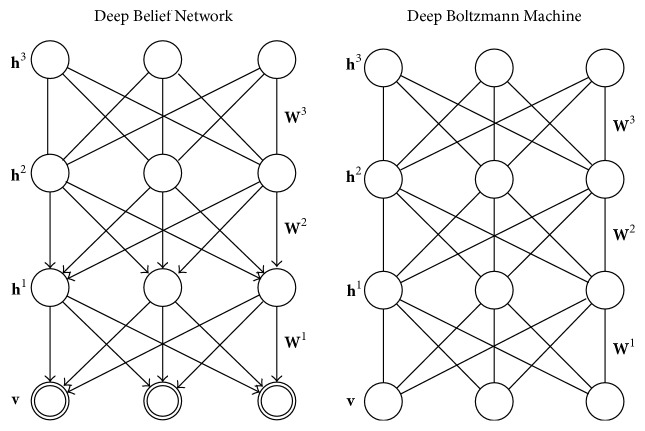
Deep Belief Network (DBN) and Deep Boltzmann Machine (DBM). The top two layers of a DBN form an undirected graph and the remaining layers form a belief network with directed, top-down connections. In a DBM, all connections are undirected.

Deep Belief Networks and Deep Boltzmann Machines are deep learning models that belong in the “Boltzmann family,” in the sense that they utilize the Restricted Boltzmann Machine (RBM) as learning module. The Restricted Boltzmann Machine (RBM) is a generative stochastic neural network. DBNs have undirected connections at the top two layers which form an RBM and directed connections to the lower layers. DBMs have undirected connections between all layers of the network. A graphic depiction of DBNs and DBMs can be found in Figure 2. In the following subsections, we will describe the basic characteristics of DBNs and DBMs, after presenting their basic building block, the RBM.

#### 2.2.1. Restricted Boltzmann Machines

A Restricted Boltzmann Machine ([34, 35]) is an undirected graphical model with stochastic visible variables **v** ∈ {0, 1}^*D*^ and stochastic hidden variables **h** ∈ {0, 1}^*F*^, where each visible variable is connected to each hidden variable. An RBM is a variant of the Boltzmann Machine, with the restriction that the visible units and hidden units must form a bipartite graph. This restriction allows for more efficient training algorithms, in particular the gradient-based contrastive divergence algorithm [36].

The model defines the energy function *E*: {0, 1}^*D*^ × {0, 1}^*F*^ → *ℝ*:

(5) $$\begin{array}{c} E\left(\;v,h;\theta \;\right)=-\overset{D}{\underset{i=1}{\sum }}\;\overset{F}{\underset{j=1}{\sum }}W_{ij}v_{i}h_{j}-\overset{D}{\underset{i=1}{\sum }}b_{i}v_{i}-\overset{F}{\underset{j=1}{\sum }}\alpha_{j}h_{j}, \end{array}$$

where *θ* = {**a**, **b**, **W**} are the model parameters; that is, *W*_*ij*_ represents the symmetric interaction term between visible unit *i* and hidden unit *j*, and *b*_*i*_, *a*_*j*_ are bias terms.

The joint distribution over the visible and hidden units is given by

(6) Pv,h;θ=1Zθexp⁡−Ev,h;θ,Zθ=∑v∑hexp⁡−Ev,h;θ,

where *𝒵*(*θ*) is the normalizing constant. The conditional distributions over hidden **h** and visible **v** vectors can be derived by (5) and (6) as

(7) Ph ∣ v;θ=∏j=1Fphj ∣ v,Pv ∣ h;θ=∏i=1Dpvi ∣ h.

Given a set of observations {**v**_*n*_}_*n*=1_^*N*^ the derivative of the log-likelihood with respect to the model parameters can be derived by (6) as

(8) $$\begin{array}{c} \frac{1}{N}\overset{N}{\underset{n=1}{\sum }}\frac{\partial \log P\left(v_{n};\theta \right)}{{\partial W}_{ij}}=E_{P_{data}}\left[\;v_{i}h_{j}\;\right]-E_{P_{model}}\left[\;v_{i}h_{j}\;\right], \end{array}$$

where *𝔼*_*P*_data__ denotes an expectation with respect to the data distribution *P*_data_(**h**, **v**; *θ*) = *P*(**h**∣**v**; *θ*)*P*_data_(**v**), with *P*_data_(**v**) = (1/*N*)∑_*n*_ *δ*(**v** − **v**_**n**_) representing the empirical distribution and *𝔼*_*P*model_ is an expectation with respect to the distribution defined by the model, as in (6).

A detailed explanation along with the description of a practical way to train RBMs was given in [37], whereas [38] discusses the main difficulties of training RBMs and their underlying reasons and proposes a new algorithm with an adaptive learning rate and an enhanced gradient, so as to address the aforementioned difficulties.

#### 2.2.2. Deep Belief Networks

Deep Belief Networks (DBNs) are probabilistic generative models which provide a joint probability distribution over observable data and labels. They are formed by stacking RBMs and training them in a greedy manner, as was proposed in [39]. A DBN initially employs an efficient layer-by-layer greedy learning strategy to initialize the deep network, and, in the sequel, fine-tunes all weights jointly with the desired outputs. DBNs are graphical models which learn to extract a deep hierarchical representation of the training data. They model the joint distribution between observed vector **x** and the *l* hidden layers **h** ^*k*^ as follows:

(9) $$\begin{array}{c} P\left(\;x,h^{1},\ldots ,h^{l}\;\right)=\left(\;\overset{l-2}{\underset{k=0}{\prod }}P\left(\;h^{k}\;|\;h^{k+1}\;\right)\;\right)P\left(\;h^{l-1},h^{l}\;\right), \end{array}$$

where **x** = **h** ^0^, *P*(**h** ^*k*^∣**h** ^*k*+1^) is a conditional distribution for the visible units at level *k* conditioned on the hidden units of the RBM at level *k* + 1, and *P*(**h** ^*l*−1^∣**h** ^*l*^) is the visible-hidden joint distribution in the top-level RBM.

The principle of greedy layer-wise unsupervised training can be applied to DBNs with RBMs as the building blocks for each layer [33, 39]. A brief description of the process follows:

1. Train the first layer as an RBM that models the raw input **x** = **h** ^0^ as its visible layer.
2. Use that first layer to obtain a representation of the input that will be used as data for the second layer. Two common solutions exist. This representation can be chosen as being the mean activation *P*(**h** ^1^ = 1∣**h** ^0^) or samples of *P*(**h** ^1^∣**h** ^0^).
3. Train the second layer as an RBM, taking the transformed data (samples or mean activation) as training examples (for the visible layer of that RBM).
4. Iterate steps ((2) and (3)) for the desired number of layers, each time propagating upward either samples or mean values.
5. Fine-tune all the parameters of this deep architecture with respect to a proxy for the DBN log- likelihood, or with respect to a supervised training criterion (after adding extra learning machinery to convert the learned representation into supervised predictions, e.g., a linear classifier).

There are two main advantages in the above-described greedy learning process of the DBNs [40]. First, it tackles the challenge of appropriate selection of parameters, which in some cases can lead to poor local optima, thereby ensuring that the network is appropriately initialized. Second, there is no requirement for labelled data since the process is unsupervised. Nevertheless, DBNs are also plagued by a number of shortcomings, such as the computational cost associated with training a DBN and the fact that the steps towards further optimization of the network based on maximum likelihood training approximation are unclear [41]. Furthermore, a significant disadvantage of DBNs is that they do not account for the two-dimensional structure of an input image, which may significantly affect their performance and applicability in computer vision and multimedia analysis problems. However, a later variation of the DBN, the Convolutional Deep Belief Network (CDBN) ([42, 43]), uses the spatial information of neighboring pixels by introducing convolutional RBMs, thus producing a translation invariant generative model that successfully scales when it comes to high dimensional images, as is evidenced in [44].

#### 2.2.3. Deep Boltzmann Machines

Deep Boltzmann Machines (DBMs) [45] are another type of deep model using RBM as their building block. The difference in architecture of DBNs is that, in the latter, the top two layers form an undirected graphical model and the lower layers form a directed generative model, whereas in the DBM all the connections are undirected. DBMs have multiple layers of hidden units, where units in odd-numbered layers are conditionally independent of even-numbered layers, and vice versa. As a result, inference in the DBM is generally intractable. Nonetheless, an appropriate selection of interactions between visible and hidden units can lead to more tractable versions of the model. During network training, a DBM jointly trains all layers of a specific unsupervised model, and instead of maximizing the likelihood directly, the DBM uses a stochastic maximum likelihood (SML) [46] based algorithm to maximize the lower bound on the likelihood. Such a process would seem vulnerable to falling in poor local minima [45], leaving several units effectively dead. Instead, a greedy layer-wise training strategy was proposed [47], which essentially consists in pretraining the layers of the DBM, similarly to DBN, namely, by stacking RBMs and training each layer to independently model the output of the previous layer, followed by a final joint fine-tuning.

Regarding the advantages of DBMs, they can capture many layers of complex representations of input data and they are appropriate for unsupervised learning since they can be trained on unlabeled data, but they can also be fine-tuned for a particular task in a supervised fashion. One of the attributes that sets DBMs apart from other deep models is that the approximate inference process of DBMs includes, apart from the usual bottom-up process, a top-down feedback, thus incorporating uncertainty about inputs in a more effective manner. Furthermore, in DBMs, by following the approximate gradient of a variational lower bound on the likelihood objective, one can jointly optimize the parameters of all layers, which is very beneficial especially in cases of learning models from heterogeneous data originating from different modalities [48].

As far as the drawbacks of DBMs are concerned, one of the most important ones is, as mentioned above, the high computational cost of inference, which is almost prohibitive when it comes to joint optimization in sizeable datasets. Several methods have been proposed to improve the effectiveness of DBMs. These include accelerating inference by using separate models to initialize the values of the hidden units in all layers [47, 49], or other improvements at the pretraining stage [50, 51] or at the training stage [52, 53].

### 2.3. Stacked (Denoising) Autoencoders

Stacked Autoencoders use the autoencoder as their main building block, similarly to the way that Deep Belief Networks use Restricted Boltzmann Machines as component. It is therefore important to briefly present the basics of the autoencoder and its denoising version, before describing the deep learning architecture of Stacked (Denoising) Autoencoders.

#### 2.3.1. Autoencoders

An autoencoder is trained to encode the input **x** into a representation **r**(**x**) in a way that input can be reconstructed from **r**(**x**) [33]. The target output of the autoencoder is thus the autoencoder input itself. Hence, the output vectors have the same dimensionality as the input vector. In the course of this process, the reconstruction error is being minimized, and the corresponding code is the learned feature. If there is one linear hidden layer and the mean squared error criterion is used to train the network, then the *k* hidden units learn to project the input in the span of the first *k* principal components of the data [54]. If the hidden layer is nonlinear, the autoencoder behaves differently from PCA, with the ability to capture multimodal aspects of the input distribution [55]. The parameters of the model are optimized so that the average reconstruction error is minimized. There are many alternatives to measure the reconstruction error, including the traditional squared error:

(10) $$\begin{array}{c} L={\left\|\;x-f\left(\;r\left(\;x\;\right)\;\right)\;\right\|}^{2}, \end{array}$$

where function **f** is the* decoder* and **f**(**r**(**x**)) is the reconstruction produced by the model.

If the input is interpreted as bit vectors or vectors of bit probabilities, then the loss function of the reconstruction could be represented by cross-entropy; that is,

(11) $$\begin{array}{c} L=-\underset{i}{\sum }x_{i}\log f_{i}\left(\;r\left(\;x\;\right)\;\right)+\left(\;1-x_{i}\;\right)\log\left(\;1-f_{i}\left(\;r\left(\;x\;\right)\;\right)\;\right). \end{array}$$

The goal is for the representation (or* code*) **r**(**x**) to be a distributed representation that manages to capture the coordinates along the main variations of the data, similarly to the principle of Principal Components Analysis (PCA). Given that **r**(**x**) is not lossless, it is impossible for it to constitute a successful compression for all input **x**. The aforementioned optimization process results in low reconstruction error on test examples from the same distribution as the training examples but generally high reconstruction error on samples arbitrarily chosen from the input space.

#### 2.3.2. Denoising Autoencoders

**Figure 3:**
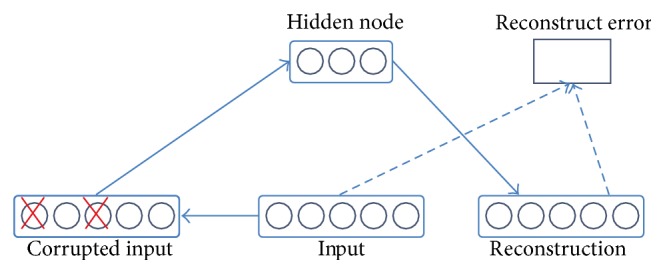
Denoising autoencoder [56].

The denoising autoencoder [56] is a stochastic version of the autoencoder where the input is stochastically corrupted, but the uncorrupted input is still used as target for the reconstruction. In simple terms, there are two main aspects in the function of a denoising autoencoder: first it tries to encode the input (namely, preserve the information about the input), and second it tries to undo the effect of a corruption process stochastically applied to the input of the autoencoder (see Figure 3). The latter can only be done by capturing the statistical dependencies between the inputs. It can be shown that the denoising autoencoder maximizes a lower bound on the log-likelihood of a generative model.

In [56], the stochastic corruption process arbitrarily sets a number of inputs to zero. Then the denoising autoencoder is trying to predict the corrupted values from the uncorrupted ones, for randomly selected subsets of missing patterns. In essence, the ability to predict any subset of variables from the remaining ones is a sufficient condition for completely capturing the joint distribution between a set of variables. It should be mentioned that using autoencoders for denoising was introduced in earlier works (e.g., [57]), but the substantial contribution of [56] lies in the demonstration of the successful use of the method for unsupervised pretraining of a deep architecture and in linking the denoising autoencoder to a generative model.

#### 2.3.3. Stacked (Denoising) Autoencoders

It is possible to stack denoising autoencoders in order to form a deep network by feeding the latent representation (output code) of the denoising autoencoder of the layer below as input to the current layer. The unsupervised pretraining of such an architecture is done one layer at a time. Each layer is trained as a denoising autoencoder by minimizing the error in reconstructing its input (which is the output code of the previous layer). When the first *k* layers are trained, we can train the (*k* + 1)th layer since it will then be possible compute the latent representation from the layer underneath.

When pretraining of all layers is completed, the network goes through a second stage of training called fine-tuning. Here supervised fine-tuning is considered when the goal is to optimize prediction error on a supervised task. To this end, a logistic regression layer is added on the output code of the output layer of the network. The derived network is then trained like a multilayer perceptron, considering only the encoding parts of each autoencoder at this point. This stage is supervised, since the target class is taken into account during training.

As is easily seen, the principle for training stacked autoencoders is the same as the one previously described for Deep Belief Networks, but using autoencoders instead of Restricted Boltzmann Machines. A number of comparative experimental studies show that Deep Belief Networks tend to outperform stacked autoencoders ([58, 59]), but this is not always the case, especially when DBNs are compared to Stacked Denoising Autoencoders [56].

One strength of autoencoders as the basic unsupervised component of a deep architecture is that, unlike with RBMs, they allow almost any parametrization of the layers, on condition that the training criterion is continuous in the parameters. In contrast, one of the shortcomings of SAs is that they do not correspond to a generative model, when with generative models like RBMs and DBNs, samples can be drawn to check the outputs of the learning process.

### 2.4. Discussion

Some of the strengths and limitations of the presented deep learning models were already discussed in the respective subsections. In an attempt to compare these models (for a summary see Table 2), we can say that CNNs have generally performed better than DBNs in current literature on benchmark computer vision datasets such as MNIST. In cases where the input is nonvisual, DBNs often outperform other models, but the difficulty in accurately estimating joint probabilities as well as the computational cost in creating a DBN constitutes drawbacks. A major positive aspect of CNNs is “feature learning,” that is, the bypassing of handcrafted features, which are necessary for other types of networks; however, in CNNs features are automatically learned. On the other hand, CNNs rely on the availability of ground truth, that is, labelled training data, whereas DBNs/DBMs and SAs do not have this limitation and can work in an unsupervised manner. On a different note, one of the disadvantages of autoencoders lies in the fact that they could become ineffective if errors are present in the first layers. Such errors may cause the network to learn to reconstruct the average of the training data. Denoising autoencoders [56], however, can retrieve the correct input from a corrupted version, thus leading the network to grasp the structure of the input distribution. In terms of the efficiency of the training process, only in the case of SAs is real-time training possible, whereas CNNs and DBNs/DBMs training processes are time-consuming. Finally, one of the strengths of CNNs is the fact that they can be invariant to transformations such as translation, scale, and rotation. Invariance to translation, rotation, and scale is one of the most important assets of CNNs, especially in computer vision problems, such as object detection, because it allows abstracting an object's identity or category from the specifics of the visual input (e.g., relative positions/orientation of the camera and the object), thus enabling the network to effectively recognize a given object in cases where the actual pixel values on the image can significantly differ.

## 3. Applications in Computer Vision

In this section, we survey works that have leveraged deep learning methods to address key tasks in computer vision, such as object detection, face recognition, action and activity recognition, and human pose estimation.

### 3.1. Object Detection

**Figure 4:**
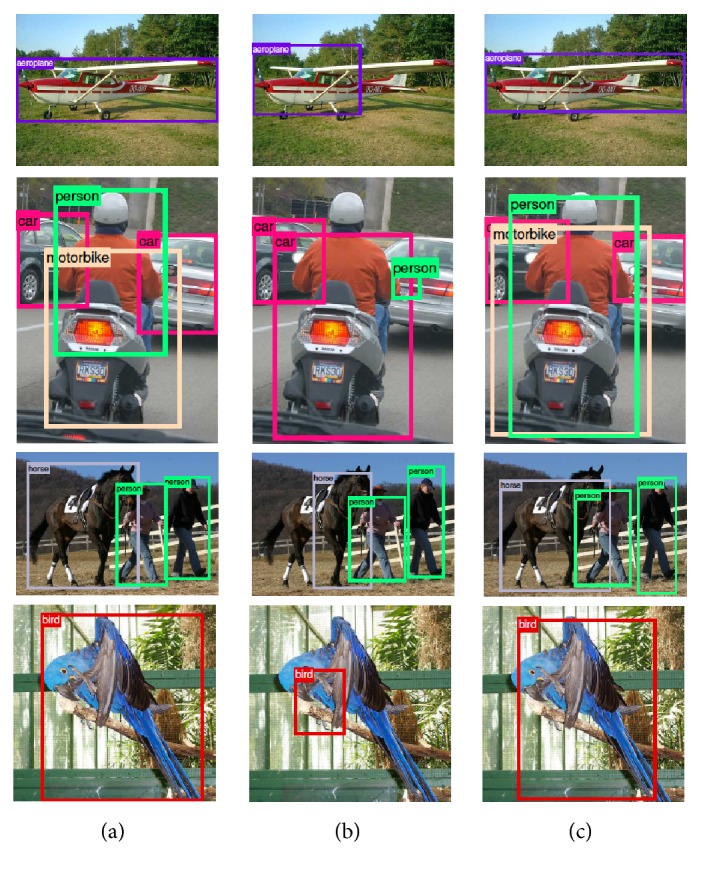
Object detection results comparison from [66]. (a) Ground truth; (b) bounding boxes obtained with [32]; (c) bounding boxes obtained with [66].

Object detection is the process of detecting instances of semantic objects of a certain class (such as humans, airplanes, or birds) in digital images and video (Figure 4). A common approach for object detection frameworks includes the creation of a large set of candidate windows that are in the sequel classified using CNN features. For example, the method described in [32] employs selective search [60] to derive object proposals, extracts CNN features for each proposal, and then feeds the features to an SVM classifier to decide whether the windows include the object or not. A large number of works is based on the concept of Regions with CNN features proposed in [32]. Approaches following the Regions with CNN paradigm usually have good detection accuracies (e.g., [61, 62]); however, there is a significant number of methods trying to further improve the performance of Regions with CNN approaches, some of which succeed in finding approximate object positions but often cannot precisely determine the exact position of the object [63]. To this end, such methods often follow a joint object detection—semantic segmentation approach [64–66], usually attaining good results.

A vast majority of works on object detection using deep learning apply a variation of CNNs, for example, [8, 67, 68] (in which a new def-pooling layer and new learning strategy are proposed), [9] (weakly supervised cascaded CNNs), and [69] (subcategory-aware CNNs). However, there does exist a relatively small number of object detection attempts using other deep models. For example, [70] proposes a coarse object locating method based on a saliency mechanism in conjunction with a DBN for object detection in remote sensing images; [71] presents a new DBN for 3D object recognition, in which the top-level model is a third-order Boltzmann machine, trained using a hybrid algorithm that combines both generative and discriminative gradients; [72] employs a fused deep learning approach, while [73] explores the representation capabilities of a deep model in a semisupervised paradigm. Finally, [74] leverages stacked autoencoders for multiple organ detection in medical images, while [75] exploits saliency-guided stacked autoencoders for video-based salient object detection.

### 3.2. Face Recognition

Face recognition is one of the hottest computer vision applications with great commercial interest as well. A variety of face recognition systems based on the extraction of handcrafted features have been proposed [76–79]; in such cases, a feature extractor extracts features from an aligned face to obtain a low-dimensional representation, based on which a classifier makes predictions. CNNs brought about a change in the face recognition field, thanks to their feature learning and transformation invariance properties. The first work employing CNNs for face recognition was [80]; today light CNNs [81] and VGG Face Descriptor [82] are among the state of the art. In [44] a Convolutional DBN achieved a great performance in face verification.

Moreover, Google's FaceNet [83] and Facebook's DeepFace [84] are both based on CNNs. DeepFace [84] models a face in 3D and aligns it to appear as a frontal face. Then, the normalized input is fed to a single convolution-pooling-convolution filter, followed by three locally connected layers and two fully connected layers used to make final predictions. Although DeepFace attains great performance rates, its representation is not easy to interpret because the faces of the same person are not necessarily clustered during the training process. On the other hand, FaceNet defines a triplet loss function on the representation, which makes the training process learn to cluster the face representation of the same person. Furthermore, CNNs constitute the core of OpenFace [85], an open-source face recognition tool, which is of comparable (albeit a little lower) accuracy, is open-source, and is suitable for mobile computing, because of its smaller size and fast execution time.

### 3.3. Action and Activity Recognition

Human action and activity recognition is a research issue that has received a lot of attention from researchers [86, 87]. Many works on human activity recognition based on deep learning techniques have been proposed in the literature in the last few years [88]. In [89] deep learning was used for complex event detection and recognition in video sequences: first, saliency maps were used for detecting and localizing events, and then deep learning was applied to the pretrained features for identifying the most important frames that correspond to the underlying event. In [90] the authors successfully employ a CNN-based approach for activity recognition in beach volleyball, similarly to the approach of [91] for event classification from large-scale video datasets; in [92], a CNN model is used for activity recognition based on smartphone sensor data. The authors of [12] incorporate a radius–margin bound as a regularization term into the deep CNN model, which effectively improves the generalization performance of the CNN for activity classification. In [13], the authors scrutinize the applicability of CNN as joint feature extraction and classification model for fine-grained activities; they find that due to the challenges of large intraclass variances, small interclass variances, and limited training samples per activity, an approach that directly uses deep features learned from ImageNet in an SVM classifier is preferable.

Driven by the adaptability of the models and by the availability of a variety of different sensors, an increasingly popular strategy for human activity recognition consists in fusing multimodal features and/or data. In [93], the authors mixed appearance and motion features for recognizing group activities in crowded scenes collected from the web. For the combination of the different modalities, the authors applied multitask deep learning. The work of [94] explores combination of heterogeneous features for complex event recognition. The problem is viewed as two different tasks: first, the most informative features for recognizing events are estimated, and then the different features are combined using an AND/OR graph structure. There is also a number of works combining more than one type of model, apart from several data modalities. In [95], the authors propose a multimodal multistream deep learning framework to tackle the egocentric activity recognition problem, using both the video and sensor data and employing a dual CNNs and Long Short-Term Memory architecture. Multimodal fusion with a combined CNN and LSTM architecture is also proposed in [96]. Finally, [97] uses DBNs for activity recognition using input video sequences that also include depth information.

### 3.4. Human Pose Estimation

The goal of human pose estimation is to determine the position of human joints from images, image sequences, depth images, or skeleton data as provided by motion capturing hardware [98]. Human pose estimation is a very challenging task owing to the vast range of human silhouettes and appearances, difficult illumination, and cluttered background. Before the era of deep learning, pose estimation was based on detection of body parts, for example, through pictorial structures [99].

Moving on to deep learning methods in human pose estimation, we can group them into holistic and part-based methods, depending on the way the input images are processed. The holistic processing methods tend to accomplish their task in a global fashion and do not explicitly define a model for each individual part and their spatial relationships. DeepPose [14] is a holistic model that formulates the human pose estimation method as a joint regression problem and does not explicitly define the graphical model or part detectors for the human pose estimation. Nevertheless, holistic-based methods tend to be plagued by inaccuracy in the high-precision region due to the difficulty in learning direct regression of complex pose vectors from images.

On the other hand, the part-based processing methods focus on detecting the human body parts individually, followed by a graphic model to incorporate the spatial information. In [15], the authors, instead of training the network using the whole image, use the local part patches and background patches to train a CNN, in order to learn conditional probabilities of the part presence and spatial relationships. In [100] the approach trains multiple smaller CNNs to perform independent binary body-part classification, followed with a higher-level weak spatial model to remove strong outliers and to enforce global pose consistency. Finally, in [101], a multiresolution CNN is designed to perform heat-map likelihood regression for each body part, followed with an implicit graphic model to further promote joint consistency.

### 3.5. Datasets

The applicability of deep learning approaches has been evaluated on numerous datasets, whose content varied greatly, according the application scenario. Regardless of the investigated case, the main application domain is (natural) images. A brief description of utilized datasets (traditional and new ones) for benchmarking purposes is provided below.

*(1) Grayscale Images.* The most used grayscale images dataset is MNIST [20] and its variations, that is, NIST and perturbed NIST. The application scenario is the recognition of handwritten digits.

*(2) RGB Natural Images.* Caltech RGB image datasets [102], for example, Caltech 101/Caltech 256 and the Caltech Silhouettes, contain pictures of objects belonging to 101/256 categories. CIFAR datasets [103] consist of thousands of 32 × 32 color images in various classes. COIL datasets [104] consist of different objects imaged at every angle in a 360 rotation. 

*(3) Hyperspectral Images.* SCIEN hyperspectral image data [105] and AVIRIS sensor based datasets [106], for example, contain hyperspectral images. 

*(4) Facial Characteristics Images.* Adience benchmark dataset [107] can be used for facial attributes identification, that is, age and gender, from images of faces. Face recognition in unconstrained environments [108] is another commonly used dataset. 

*(5) Medical Images.* Chest X-ray dataset [109] comprises 112120 frontal-view X-ray images of 30805 unique patients with the text-mined fourteen disease image labels (where each image can have multilabels). Lymph Node Detection and Segmentation datasets [110] consist of Computed Tomography images of the mediastinum and abdomen. 

*(6) Video Streams.* The WR datasets [111, 112] can be used for video-based activity recognition in assembly lines [113], containing sequences of 7 categories of industrial tasks. YouTube-8M [114] is a dataset of 8 million YouTube video URLs, along with video-level labels from a diverse set of 4800 Knowledge Graph entities.

## 4. Conclusions

The surge of deep learning over the last years is to a great extent due to the strides it has enabled in the field of computer vision. The three key categories of deep learning for computer vision that have been reviewed in this paper, namely, CNNs, the “Boltzmann family” including DBNs and DBMs, and SdAs, have been employed to achieve significant performance rates in a variety of visual understanding tasks, such as object detection, face recognition, action and activity recognition, human pose estimation, image retrieval, and semantic segmentation. However, each category has distinct advantages and disadvantages. CNNs have the unique capability of feature learning, that is, of automatically learning features based on the given dataset. CNNs are also invariant to transformations, which is a great asset for certain computer vision applications. On the other hand, they heavily rely on the existence of labelled data, in contrast to DBNs/DBMs and SdAs, which can work in an unsupervised fashion. Of the models investigated, both CNNs and DBNs/DBMs are computationally demanding when it comes to training, whereas SdAs can be trained in real time under certain circumstances.

As a closing note, in spite of the promising—in some cases impressive—results that have been documented in the literature, significant challenges do remain, especially as far as the theoretical groundwork that would clearly explain the ways to define the optimal selection of model type and structure for a given task or to profoundly comprehend the reasons for which a specific architecture or algorithm is effective in a given task or not. These are among the most important issues that will continue to attract the interest of the machine learning research community in the years to come.

## Figures and Tables

**Table 1:** Important milestones in the history of neural networks and machine learning, leading up to the era of deep learning.

Milestone/contribution	Contributor, year
MCP model, regarded as the ancestor of the Artificial Neural Network	McCulloch & Pitts, 1943
Hebbian learning rule	Hebb, 1949
First perceptron	Rosenblatt, 1958
Backpropagation	Werbos, 1974
Neocognitron, regarded as the ancestor of the Convolutional Neural Network	Fukushima, 1980
Boltzmann Machine	Ackley, Hinton & Sejnowski, 1985
Restricted Boltzmann Machine (initially known as Harmonium)	Smolensky, 1986
Recurrent Neural Network	Jordan, 1986
Autoencoders	Rumelhart, Hinton & Williams, 1986
	Ballard, 1987
LeNet, starting the era of Convolutional Neural Networks	LeCun, 1990
LSTM	Hochreiter & Schmidhuber, 1997
Deep Belief Network, ushering the “age of deep learning”	Hinton, 2006
Deep Boltzmann Machine	Salakhutdinov & Hinton, 2009
AlexNet, starting the age of CNN used for ImageNet classification	Krizhevsky, Sutskever, & Hinton, 2012

**Table 2:** Comparison of CNNs, DBNs/DBMs, and SdAs with respect to a number of properties. + denotes a good performance in the property and − denotes bad performance or complete lack thereof.

Model properties	CNNs	DBNs/DBMs	SdAs
Unsupervised learning	−	+	+
Training efficiency	−	−	+
Feature learning	+	−	−
Scale/rotation/translation invariance	+	−	−
Generalization	+	+	+
